# The Core Mechanism of Yiqi Yangjing Decoction Inhibiting Nonsmall-Cell Lung Cancer

**DOI:** 10.1155/2022/2256671

**Published:** 2022-05-09

**Authors:** Kaiyan Yi, Yaning Zhou, Ming Zhang, Yijun Guo

**Affiliations:** Shanghai Chest Hospital, Shanghai Jiao Tong University, Shanghai 200030, China

## Abstract

**Background:**

Yiqi Yangjing prescription (YQYJ) is a traditional Chinese medicine prescription used for treating lung cancer. It has a significant effect on enhancing efficacy, reducing toxic symptoms, and improving patients' physical well-being. The effective inhibitory effect on nonsmall-cell lung cancer (NSCLC) has been demonstrated *in vitro* and *in vivo*. However, the mechanism of action and the material basis still remain unclear.

**Methods:**

In this study, we explored this mechanism using network pharmacology, after which we explored the pharmacodynamics and the action mechanism of YQYJ using cell viability evaluation, plate clone formation assay, flow cytometry, real-time quantitative PCR, and Western blot.

**Results:**

The enrichment results showed that there were 50 active components and 68 core targets related to YQYJ inhibiting NSCLC, including quercetin, luteolin, gamatin, kaempferol, heat shock protein HSP 90-alpha (HSP90AA1), cyclin-dependent kinase 2 (CDK2), epidermal growth factor receptor (EGFR), signal transducer and activator of transcription 3 (STAT3), and others. Among them, quercetin and kaempferol revealed the best binding effect with core targets. Most importantly, YQYJ promoted A549 cells from the quiescent phase into the proliferative phase to enhance the sensitivity of A549 cells to YQYJ and inhibited the proliferation of A549 cells significantly (*P* < 0.05). The A549 cells were blocked in both S and G2/M phases while the apoptosis ratio was increased. The proliferation score of A549 cells treated with YQYJ was significantly reduced compared to A549 cells in the proliferative phase (*P* < 0.05). This regulatory effect was related to the expression regulation of HSP90AA1, CDK2, STAT3, and phosphor-STAT3 (p-STAT3) by YQYJ, kaempferol, and quercetin.

**Conclusion:**

Our results suggested that the inhibition of NSCLC via YQYJ had multicomponent and multitarget characteristics. Its core mechanism is related to the regulation of the cell cycle, proliferation, and apoptosis of NSCLC. This study provides a direction and scientific basis for exploring the future mechanism of YQYJ for the treatment of NSCLC.

## 1. Introduction

Global cancer statistics for 2020 revealed that lung cancer accounted for 18.0% of the total cancer deaths, remaining the leading cause of cancer-related death [1]. According to previous studies, integrated therapies (e.g., radiotherapy and targeted therapy) can effectively increase survival time and quality of survival in patients with lung cancer. Traditional Chinese medicine (TCM) also has an important role in integrated therapies. Yiqi Yangjing decoction (YQYJ), also named Feiyanning, is a traditional Chinese medicine prescription used for treating lung cancer. YQYJ has obvious advantages in enhancing efficacy, reducing toxic symptoms, and improving survival quality and quality of patients' life [2–4]. It consists of “Astragali Radix,” “Ganoderma,” “Atractylodis Macrocephalae Rhizoma,” “Herb Salviae Chinensis,” “Epimedii Folium,” “Corni Fructus,” “Cremastrae Pseudobulbus,” “Polygonati Rhizoma,” “Vespae Nidus,” “Paridis Rhizoma,” and “Bufonis Venenum”. A previous study revealed that the disease control rate of the TCM combined group was slightly higher compared to the chemotherapy group, while the adverse reactions were significantly decreased [2]. Also, the experiments with mouse transplantation tumors confirmed that YQYJ could effectively inhibit the growth of NSCLC tumors and prevent the metastasis and diffusion of cancer cells [5]. Moreover, *in vitro* experiments have also led to a consistent conclusion that YQYJ has a pronounced effect on inhibiting the proliferation and migration of human NSCLC cells [6]. However, the core mechanism of YQYJ still remains unclear.

**Figure 1:**
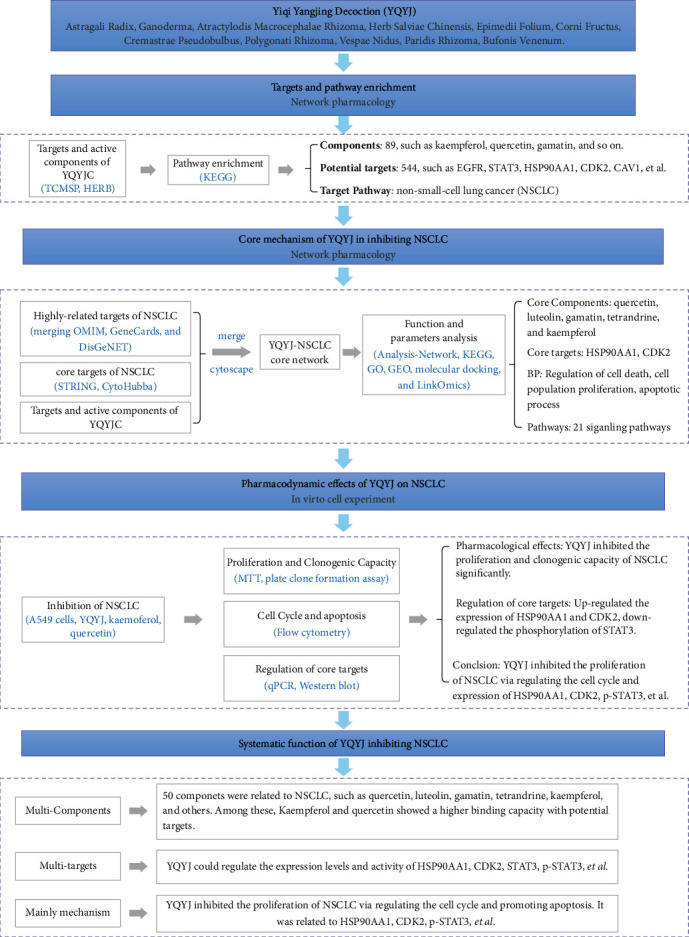
The comprehensive strategy of YQYJ inhibits NSCLC.

The development of network pharmacology and bioinformatics provides a brand-new perspective and breakthrough for explaining the scientific role of TCM [7]. The network pharmacology reveals the potential signaling pathways and provides a powerful direction for further explaining the pharmacodynamics mechanisms at the molecular level. Herein, we explored the role of active components and core targets of YQYJ in inhibiting NSCLC using network pharmacology. A comprehensive strategy of network pharmacology and multiple pharmacodynamics experiment are shown in Figure 1.

## 2. Materials and Methods

### 2.1. Component-Target Network Construction and Analysis

Identification of active components and protein targets of YQYJ: the Traditional Chinese Medicine Systems Pharmacology (TCMSP) database (https://tcmsp-e.com/) [8] and HERB database (https://herb.ac.cn/) [9] were used to screen the active components and targets of YQYJ based on ADME parameters. The HERB database, Scifinder^n^ (https://scifinder-n.cas.org/), Swiss ADME (https://www.swissadme.ch/) [10], and Swiss Target prediction database (https://www.swisstargetprediction.ch/) [11] were used to screen the active components and targets of YQYJ, which were not collected on the TCMSP platform. The screening conditions in TCMSP were oral bioavailability ≥30% and drug-likeness ≥0.18, while they were high for GI absorption in Swiss ADME. Also, to predict drug-likeness (Lipinski. Ghose, Veber, Egan, and Muegge) and potential targets, it was necessary to meet high, and three of the five rules in Swiss ADME and have a probability value >0 in the Swiss Target prediction database.

Identification of protein targets of NSCLC: the gene information of “Non-Small Cell Lung Cancer” was downloaded from the Online Mendelian Inheritance in Man (OMIM) (https://www.omim.org/), DisGeNET (https://www.disgenet.org/home/), and GeneCards (https://www.genecards.org/,%20very.5.0). Next, we screened the top lowest 10% targets of Disease Specificity Index (DSI) score in DisGeNet (evidence index = 1) and the top highest 10% of relevance score (RS) in GeneCards (RS higher than the average value of 14.88). The core targets of NSCLC were screened by using the cytoHubba plugin in Cytoscape based on the protein-protein interaction (PPI) network constructed by the STRING database (score ≥0.90) (https://cn.string-db.org/) and Cytoscape 3.8.2 software. All of the target IDs described above were standardized using the UniProt database. Analysis Network plugin estimated the topological parameters (degree) of a core network of YQYJ inhibiting NSCLC to screen the core active components and critical targets.

Gene Ontology (GO) and Kyoto Encyclopedia of Genes and Genomes (KEGG) enrichment analysis clarified the functions and potential pathways of YQYJ by using R packages (“clusterprofile,” “org.Hs.eg.db,” and “enrichment”). It was based on the *P* value cutoff = 0.05 and *q*-value cutoff = 0.05 criteria. The enrichment results were plotted by using the Sangerbox tools (https://vip.sangerbox.com/index.html), a free online platform for data analysis.

### 2.2. Molecular Docking

Processing and optimization of molecular docking were done by the Schrodinger Maestro software. The target protein crystal structures (CDK2 (PDB ID:3PY0), HSP90AA1 (PDB ID:3TUH)) were downloaded from the Protein database (https://www.rcsb.org/), and receptors were preprocessed, optimized, and minimized (constraint minimized using the OPLS3e force field) by the Protein Preparation Wizard module of Schrodinger [12, 13]. The 3D structure of active components was downloaded from the PubChem database (https://pubchem.ncbi.nlm.nih.gov/) and prepared by hydrogenation, structural optimization, and energy minimization of the LigPre module in Schrodinger. Finally, the proto-ligand was used as the active site position, and the protein was selected as the centroid of the 10 Å box for molecular docking and screening in the Glide SP computing module. The following formula was used for the calculation of the scoring function (1) [14]:

(1) $$\begin{array}{c} \Delta G_{\text{bind}}=C_{\text{lipo}-\text{lipo}\sum f\left(r_{lr}\right)}+C_{\text{hbond}-\text{neut}-\text{neut}\sum g\left(\Delta r\right)h\left(\Delta \alpha \right)}+C_{\text{hbond}-\text{neut}-\text{charged}}+C_{\text{hbond}-\text{charged}-\text{charged}\sum g\left(\Delta r\right)h\left(\Delta \alpha \right)}+C_{\max-\text{metal}-\text{ion}\sum f\left(r_{lm}\right)}+C_{\text{rotb}}H_{\text{rotb}}+C_{\text{polar}-\text{phob}}V_{\text{polar}-\text{phob}}+C_{\text{coul}}E_{\text{coul}}+C_{vdW}E_{vdW}+\text{solvationterms}. \end{array}$$

The complex formed by the docking compound and the protein was visualized using Pymol 2.1 software (the compound with the most negative binding energy for each target) to obtain the binding mode of the compound and the protein. The amino acid residues of the compound and the protein pocket could be seen according to the binding mode.

### 2.3. The Analysis of the Potential Regulatory Mechanisms of Critical Genes

Expression microarray series GSE19188 and GSE33532 were collected from the Gene Expression Omnibus data set (GEO) and used to analyze HSP90AA1, CDK2, and E2F1 expression levels in NSCLC. In addition, GSE31908 was used to analyze the relationship between the expression level of HSP90AA1 and poor overall survival (OS) in NSCLC based on Kaplan-Meier Plotter (https://kmplot.com/analysis/). Next, we explored the probable mechanisms of HSP90AA1 and CDK2 in NSCLC through LinkedOmics (https://linkedomics.org/admin.php), a platform for analyzing cancer multi-omics data based on the TCGA project [15]. Gene set enrichment analysis (GSEA) in the Link Interpreter module was used to conduct KEGG pathways, miRNA-target, and transcription factor (TF) -target enrichment and reveal potential regulatory mechanisms. The rank standard was FDR <0.05 and 500 simulations. Furthermore, we analyzed the functions of E2F1, HSP90AA1, and CDK2 in GeneMANIA (https://genemania.org/) [16] and constructed their functional interaction network.

### 2.4. Cell Culture

Human NSCLC cell line A549 was saved in the library of Shanghai Chest Hospital. Cells were cultured in RPMI 1640 medium (HyClone, Logan, Utah, USA), containing 10% fetal bovine serum (FBS) (Thermo Fisher Scientific, USA) and 1% penicillin/streptomycin, in a humidified atmosphere containing 5% CO_2_/95% air and 37°C.

### 2.5. Medicines and Reagents

Chinese Herbal Medicine YQYJ was provided by the Shanghai Institute of Materia, Chinese Academy of Sciences (Shanghai, China) (Supplementary Information S1). Kaempferol (CAS:520-18-3) and quercetin (CAS:117-39-5) were purchased from MedChemExpress (MCE, New Jersey, USA) and solubilized in DMSO. All of the stock solutions were stored at −20°C. The RPMI 1640 medium and phosphate-buffered saline (PBS) were acquired from HyClone (Logan, Utah, USA). FBS and penicillin-streptomycin solution were obtained from Gibco (Grand Island, NY, USA). Methyl thiazolyl tetrazolium (MTT), RIPA Buffer Reagent, and the BCA Protein Concentration Assay Kit were acquired from Beyotime (Shanghai, China). The primers and DMSO were purchased from Sangon Biotech (Shanghai, China). Primary antibodies against GAPDH (#8884), HSP90*α* (#8165), CDK2 (#2546), and Anti-rabbit IgG HRP-linked Antibody (#7074) were purchased from Cell Signaling Technology (Boston, USA). Primary antibodies against STAT3 (ab68153) and phosphor-STAT3 (p-STAT3) (ab76315) were obtained from Abcam (Cambridge, UK). Cell Cycle Staining Kit and Annexin V-FITC/PI apoptosis Kit were purchased from MULTI SCIENCES (Shanghai, China). The E.Z.N.A.® Total RNA kit was bought from Omega Bio-Tek (Norcross, Georgia, USA). The ChamQ® SYBR qPCR Master Mix, HiScript II Reverse Transcriptase, and ECL chemiluminescence reagents were purchased from Vazyme (Nanjing, China).

### 2.6. Cell Viability and Clone Efficiency Analysis

Cell viability and clone efficiency reflected the impact of YQYJ on the toxic effects and proliferative capacity of A549 cells. Briefly, A549 cells were inoculated with 5 × 10^3^ per well in a 96-well culture plate, and the volume of the culture medium was 100 *μ*L for 12 h. Cells were then exposed to gradually increased concentration (0.18, 0.25, 0.375, 0.5, 0.75, 1.5, and 3.0 mg/mL) of YQYJ for 12, 24, and 48 hours and gradually increased concentration (5, 10, 20, and 40 *μ*mol/mL) of kaempferol or quercetin for 24 hours. At each time point, 10 *μ*L of sterile MTT dye (5 g/L) was added to each well and incubated for another four hours at 37°C. After removal of the medium, 150 *μ*L DMSO was added to each well and properly mixed and dissolved at room temperature for another 15 min. The absorbance at 490 nm was determined using a microplate reader Synergy H1 Hybrid (Bio Tek Instruments, Germany) and Gen5 software. Cell viability was calculated using the following formulas:

(2) $$\begin{array}{c} \text{Cell viability}\left(\%\right)=\frac{\text{Experimental Group A}-\text{Blank Group A}}{\text{Control Group A}-\text{Blank Group A}}\times 100\%. \end{array}$$

For the clone efficiency experiment, A549 cells were treated with YQYJ (0.25, 0.5, and 1.0 mg/mL) for 24 hours, and then seeded 1000 per well and cultured until significant clonal formation (greater than 50 cells). Clone efficiency was measured using the following formula:

(3) $$\begin{array}{c} \text{Clone efficiency}=\frac{\text{Number of clones}}{\text{Vaccinated cells}}\times 100\%. \end{array}$$

### 2.7. Flow Cytometry

A549 cells were treated with kaempferol, quercetin, or different concentrations of YQYJ (0.25, 0.5, and 1.0 mg/mL) for 24 hours and then stained with Cell Cycle Staining Kit or Annexin V-FITC/PI apoptosis Kit (MULTI SCIENCES, Shanghai, China) according to the manufacturer's instructions. All cells were collected and then checked with BD FACSCanto II (USA). The results of the cell cycle and apoptosis ratio were calculated using ModFit LT 3.2, and the images of the apoptosis were exported using Flowjo 10.8.1.

### 2.8. Quantitative Real-Time PCR Assay (RT-qPCR)

The total RNA of each experimental group was isolated from experimental groups using E.Z.N.A.® Total RNA kit according to the manufacturer's instructions. The extracted RNA was reverse transcribed into cDNA using HiScript II Reverse Transcriptase. The qRT-PCR was conducted using an ABI viia7 real-time PCR System (Life Technologies, Loughborough, UK) and ChamQ® SYBR qPCR Master Mix. The target genes primers were designed and synthesized by Sangon Biotech (Shanghai, China). The GAPDH (the internal reference) was obtained from the primer bank. The relative gene expression was calculated using the 2^–ΔΔCT^ method as previously described. The specific experimental steps and conditions are shown in Supplementary Information S1.

### 2.9. Western Blot

The protein regulation of HSP90AA1, CDK2, STAT3, and p-STAT3 by YQYJ was estimated by Western blotting. RIPA buffer was used to extricate the total protein of experimental groups. GAPDH was used as the internal standard. Finally, the protein bands were detected using ECL chemiluminescence reagents and biomolecular imagers (Thermo Fisher Scientific, USA). The details of the experiment are shown in Supplementary Information S1.

### 2.10. Statistical Analysis

SPSS 25.0 software was used for statistical analysis. The experimental results are described with mean and standard deviation (SD). Analysis of variance (ANOVA) and Tukey's test were applied to compare the mean of each group with that of the control group. *P*-value < 0.05 was considered as statistical significance.

## 3. Results

### 3.1. Identification of Potential Targets and Pathways

**Figure 2:**
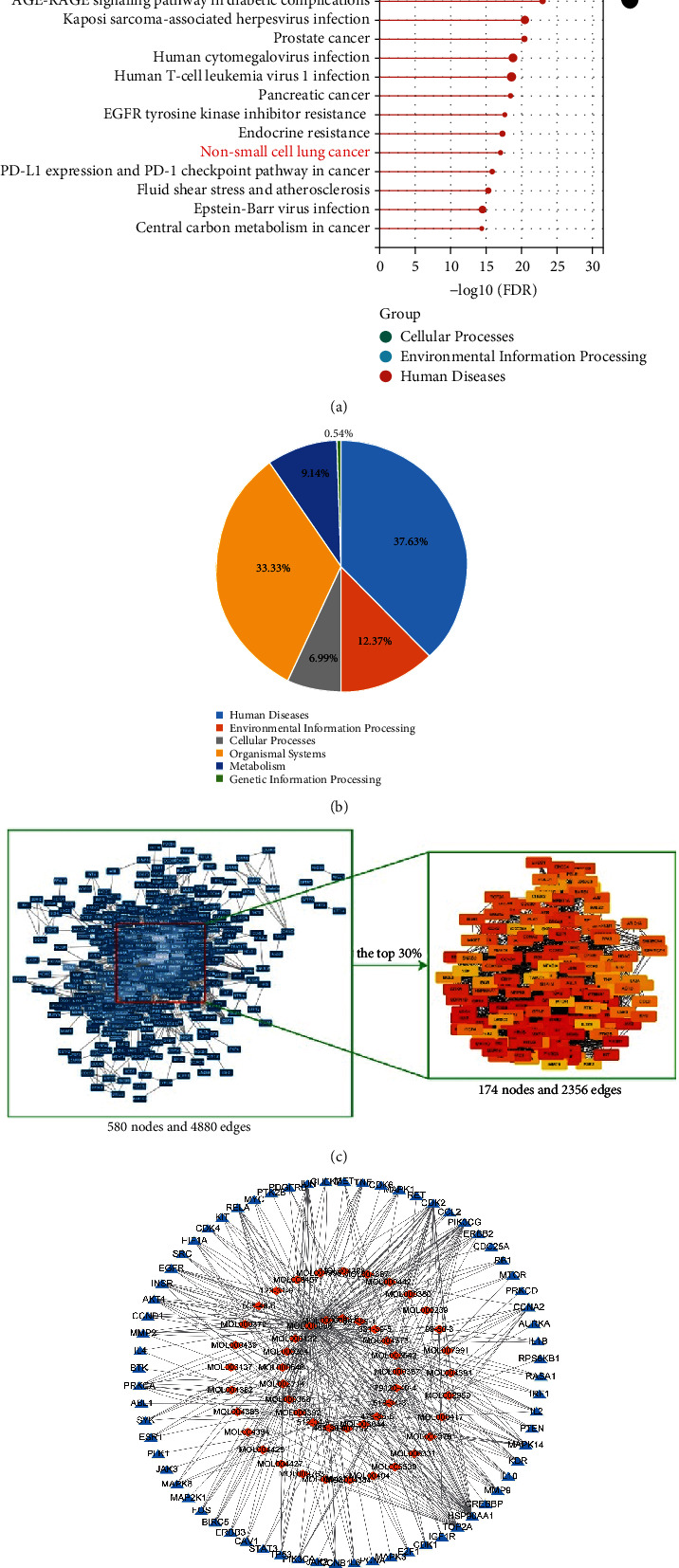
Identification of potential targets and pathways. (a) Pathway analysis based on targets of the active components in YQYJ. (b) The classification pie map of the KEGG enrichment pathway. (c) The PPI network and 174 core targets of NSCLC. (d) The core network of YQYJ inhibits NSCLC. The orange diamond node represents the 50 active components of YQYJ, and the blue triangle node represents 68 potential targets of YQYJ inhibiting NSCLC.

First, we analyzed the active components, potential targets, and action pathways of YQYJ by using network pharmacology. The active components and potential targets of YQYJ from the TCMSP database and HERB database were based on the ADME parameters, which are the classic data used for screening drug ingredients and represent the body's disposal process after the drug enters the body. There were 89 active components and 544 targets of YQYJ, as shown in Figure S1, including quercetin, kaempferol, luteolin, gamatin, signal transducer and activator of transcription 3 (STAT3), E2F Transcription Factor 1 (E2F1), epidermal growth factor receptor (EGFR), caveolin-1 (CAV1), and so on. KEGG enrichment revealed the multiple potential pathways of YQYJ (Figure 2(a)). In total, 37.63% of pathways of YQYJ were related to human disease, 12.37% were associated with environmental information processing, 6.99% were related to cellular processes, 33.33% were associated with the organismal system, and 9.14% were related to metabolism (Figure 2(b)). Most importantly, it was found that YQYJ was enriched in NSCLC (the low *P-*value and higher rich factor value in Figure 2(a)).

To further analyze the mechanism through which YQYJ inhibits NSCLC, we screened 174 core targets of NSCLC from 1093 highly related target genes of NSCLC by using the STRING database (score ≥0.900) and cytoHubba plugin of Cytoscape (Figure 2(c)). Then, a YQYJ-NSCLC core network was constructed using Cytoscape 3.8.2 software by merging the potential targets of YQYJ and NSCLC (Figure 2(d)). The orange diamond node represents the 50 active components of YQYJ, and the blue triangle node represents 68 potential targets of YQYJ inhibiting NSCLC.

**Figure 3:**
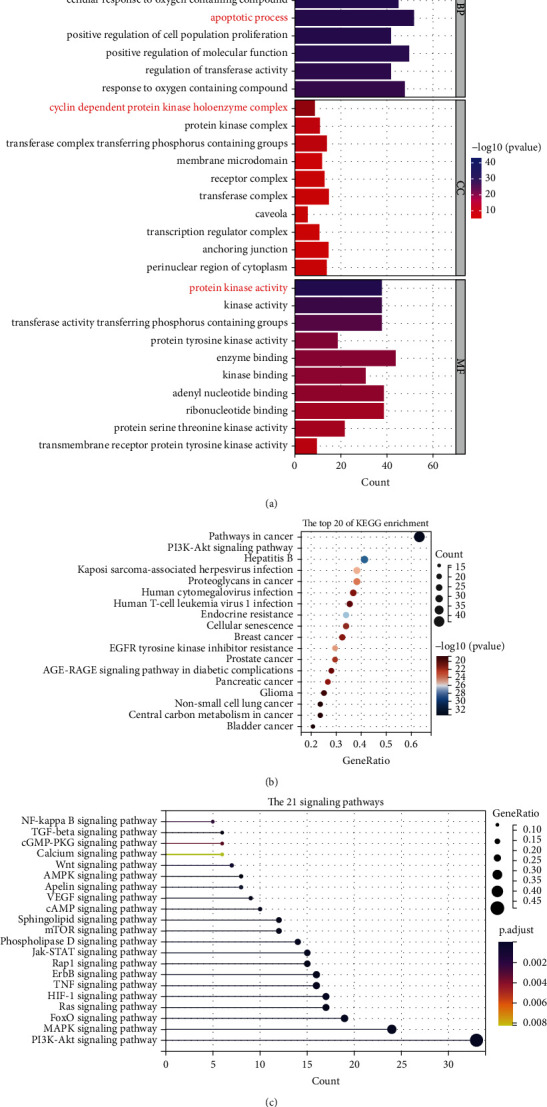
The KEGG and GO enrichment of the YQYJ-NSCLC core network. (a) GO analysis based on core targets of YQYJ inhibiting NSCLC. (b) The top 20 of KEGG enrichment of the 68 core targets of YQYJ inhibiting NSCLC. (c) The 21 signaling pathways of the YQYJ-NSCLC core network.

In this network, many active components (e.g., beta-sitosterol, tetrandrine, kaempferol, flavone, and quercetin) were found to bind one or more genes, contributing to proliferation and apoptosis (e.g., E2F1, stem cell growth factor receptor, STAT3, retinoblastoma-associated protein, and mitogen-activated protein kinase 14). Also, the functional analysis revealed that the YQYJ-NSCLC core network was involved in multiple biological processes, including protein phosphorylation, regulation of cell death, cell population proliferation, apoptotic process, protein kinase activity, nuclear factor kappa B, hypoxia-inducible factor 1 (HIF-1), phosphoinositide 3 kinase/Akt (PI3K-Akt), and Wnt, calcium signaling pathways (Figure 3). In addition, the regulatory effect of YQYJ on the HIF-1 [17], tumor necrosis factor (TNF) [18], PI3K-Akt [19], and E2F1 [20] of NSCLC cells has been already demonstrated. The uncontrolled growth properties of excessive tumor cell proliferation and reduced apoptosis have a major role in tumor progression [21]. The obtained results displayed that the components of YQYJ could intervene in NSCLC progress by binding to multiple targets and regulating cell population proliferation and death.

### 3.2. Analysis of Core Components and Targets of YQYJ

In the core network, quercetin, luteolin, tetrandrine, gamatin, and kaempferol were the top 5 disease-related components, while heat shock protein HSP 90-alpha (HSP90AA1), cyclin-dependent kinase 2(CDK2), phosphatidylinositol-4,5-bisphosphate 3-kinase, catalytic subunit gamma (PIK3CG), DNA topoisomerase 2-alpha (TOP2A), and mitogen-activated protein kinase 14 (MAPK14) were the top 5 targets (Table 1). The degree, betweenness centrality, and closeness centrality of all the above components and targets were higher than the average value. That means that they may have an important role in the core network of YQYJ inhibiting NSCLC. So, we choose the five active components and the top two targets (HSP90AA1 and CDK2) to analyze the regulation of NSCLC by YQYJ further.

**Figure 4:**
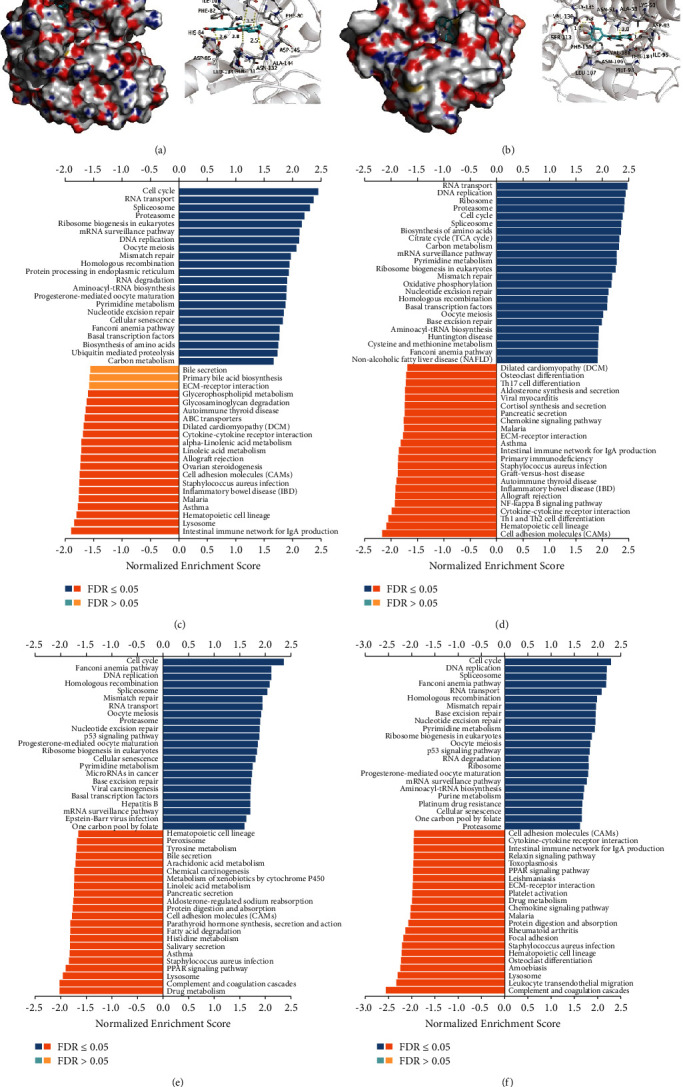
The analysis of core targets. (a) The surface and detailed mode of CDK2-quercetin. (b) The surface and detailed mode of HSP90A1-quercetin. Compounds are rendered in bright blue. Active site residues are displayed in gray. Yellow dash represents hydrogen bond distance or *π*-stacking. (c) Pathway enrichment of HSP90AA1 coexpression genes in LUAD. (d) Pathway enrichment of HSP90AA1 coexpression genes in LUSC. (e) Pathway enrichment of CDK2 coexpression genes in LUAD. (f) Pathway enrichment of CDK2 coexpression genes in LUSC.

First, we analyzed the binding potency of the active components and target proteins by molecular docking. Gemcitabine, a tumor chemotherapy drug whose intracellular metabolites bind to DNA and act primarily in the G1/S phase [22, 23], was selected for docking with the core targets. Also, the binding potency data worked as a baseline for positive control. The results shown in Table 2 indicate that compounds had an excellent binding effect with the target protein and binding energy of <6 kcal/mol. The binding energies of the four active components (quercetin, luteolin, kaempferol, and gamatin) to critical targets were lower than gemcitabine, which suggested that these components could stably bind to the active pocket of the HSP90AA1 and CDK2 protein. Among them, kaempferol and quercetin performed best in docking scoring and binding patterns with proteins (Table 2, Figures 4(a) and 4(b)). All of them showed significant inhibition of NSCLC [24–26]. For example, quercetin inhibits NSCLC proliferation and induces apoptotic through the lncRNA SNHG7/miR-34a-5p pathway [24]. Kaempferol inhibits Nrf2 signaling by inducing apoptotic in NSCLC cells by downregulation of Nrf2 mRNA [25]. Also, it effectively restores the chemorefractory phenotype relating to the EMT pathway [26].

**Figure 5:**
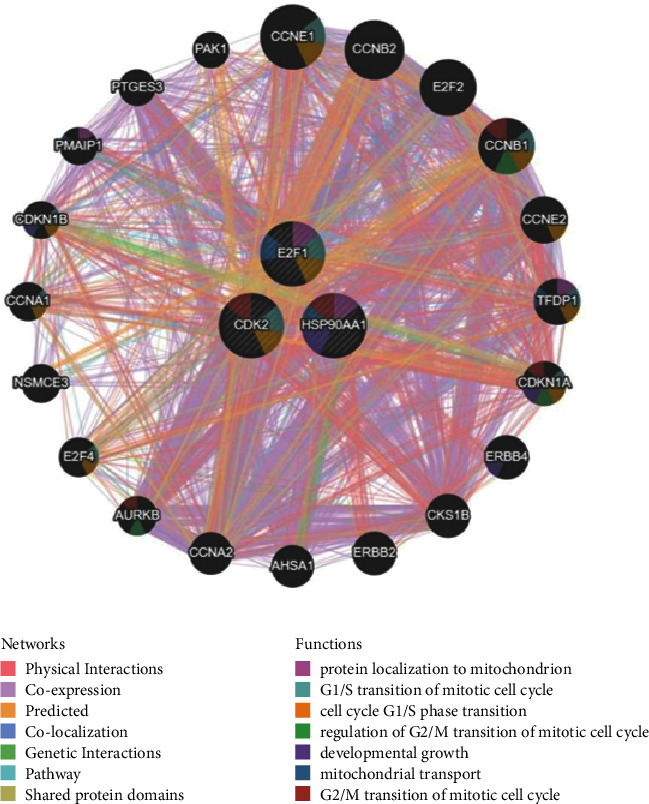
The PPI network analysis of HSP90AA1, CDK2, and E2F1 from GeneMANIA.

KEGG pathway analysis showed that HSP90AA1 and CDK2 coexpressed genes in NSCLC participate in metabolism (pyrimidine metabolism), cell growth and death (e.g., cell cycle), and genetic information processing (e.g., mRNA surveillance pathway, DNA replication, and nucleotide excision repair) (Figures 4(c)–4(f)). Higher expression of pyrimidine synthesis genes has been shown to usually result in a poor prognosis for patients with glioblastoma [27] and NSCLC [28]. Targeted pyrimidine synthesis can inhibit glioblastoma and thus can be used as a novel inhibition strategy [27]. The cell cycle, the regulation of genetic information, is closely related to tumor cell proliferation [29]. The functions of CDK2 and HSP90AA1 were also related to the G2/M transition of the mitotic cell cycle according to the PPI network from GeneMania (Figure 5). As HSP90AA1 and CDK2 were important in the cell cycle and pyrimidine metabolism, we had reason to believe that HSP90AA1 and CDK2 have a positive role in lung cancer proliferation.

**Figure 6:**
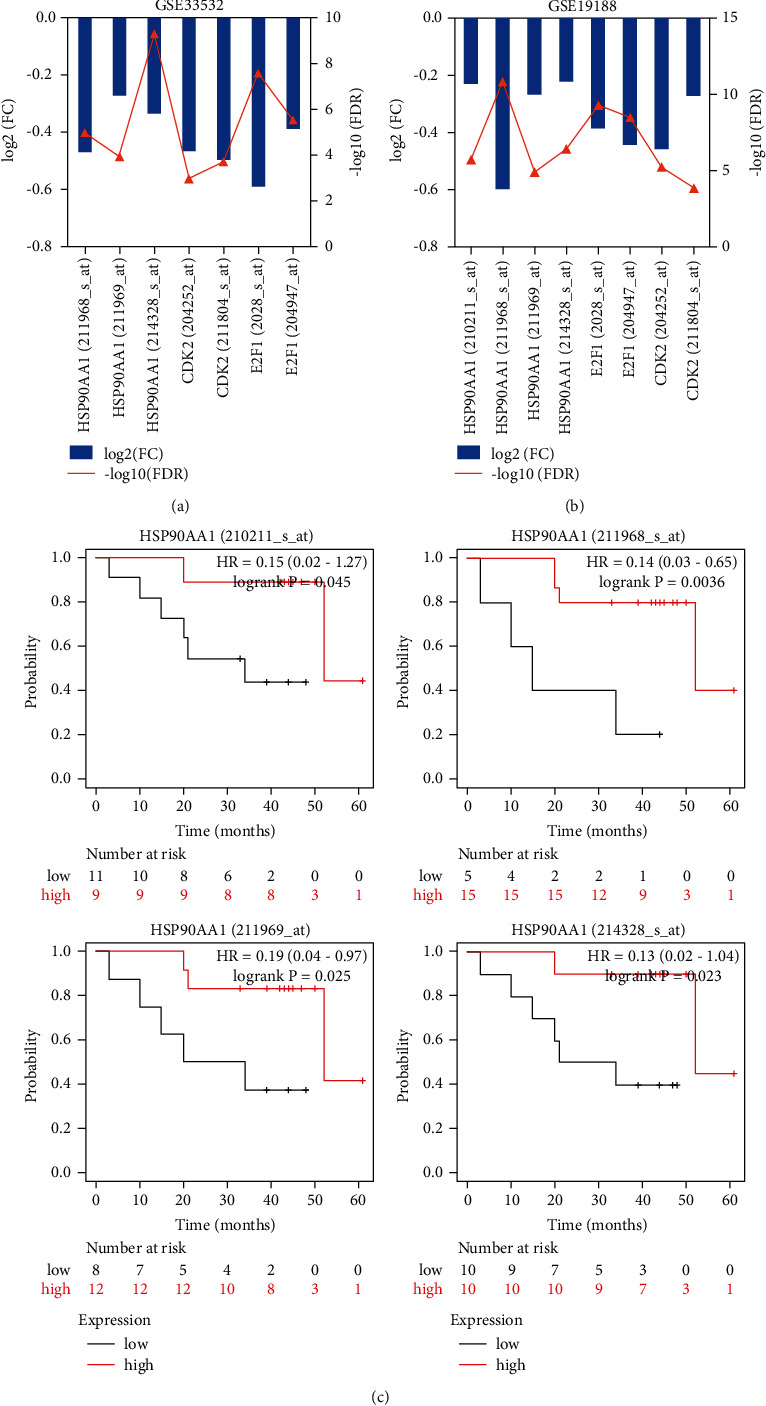
The analysis of HSP90AA1, CDK2, and E2F1 in GEO expression microarray series. (a) GSE33532. (b) GSE19188. (c) HSP90AA1 expression level with poor OS of LUAD in GSE31908.

Next, we explored the regulatory enrichment of HSP90AA1 and CKD2 coexpressed genes in NSCLC, including the enrichment of miRNAs and TF (Table 3). MIR-323 ranked first in the HSP90AA1 regulatory network of LUAD, and E2F1 ranked the highest in both the HSP90AA1 and CDK enriched networks. miR-323, which is significantly upregulated in lung cancer cells, controls A549 cell proliferation and apoptosis by regulating the AKT and ERK signaling pathways [30]. E2F1 is a transcription factor that has an important role in S phase progression and apoptosis. Although E2F1 overexpression in multiple tumors promotes tumor proliferation [31], its low expression in lung adenocarcinoma may be associated with promoting immune escape from tumor cells [32]. We also found a downregulation trend of E2F1 (log (FC) < 0 and FDR <0.05) in the data sets GSE33532 (Figure 6(a)) and GSE19188 (Figure 6(b)). The expression of CDK2 and HSP90AA1 was consistent with that of E2F1 (Figures 6(a) and 6(b)). Moreover, the low expression level of HSP90AA1 in GSE31908 was associated with poor OS (Figure 6(c)). These findings confirmed that E2F1, HSP90AA1, and CDK2 could be involved in the genetic information and cell cycle regulation in the process of NSCLC tumorigenesis.

### 3.3. Inhibitory Effect of YQYJ on a549 Cells

**Figure 7:**
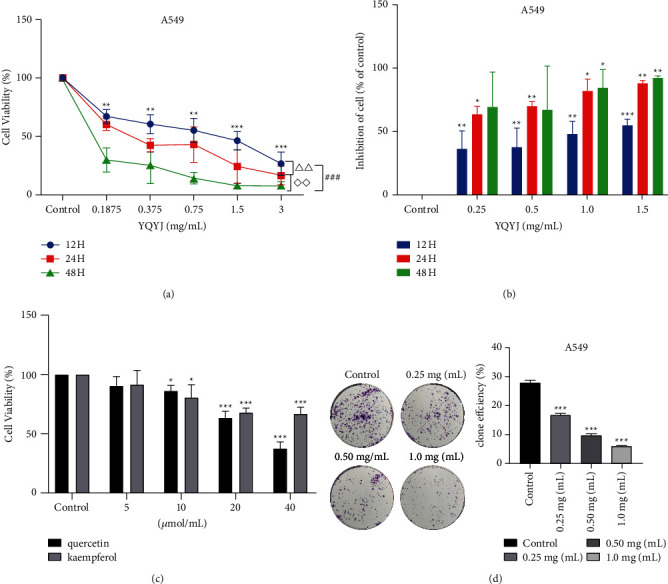
(a, b) YQYJ inhibited the proliferation of A549 cells, in a dose- and time-dependent manner (^*∗*^YQYJ groups vs control group ^*∗*^*P* < 0.05, ^*∗∗*^*P* < 0.01, ^*∗∗∗*^ *P* < 0.001; △△ 12 hours vs 24 hours *P* < 0.01; △△ 24 hours vs 48 hours *P* < 0.01; ^###^ 12 hours vs 48 hours *P* < 0.001). (c) The kaempferol (10, 20, and 40 *μ*mol/mL) and quercetin (10, 20, and 40 *μ*mol/mL) significantly inhibited the proliferation of A549 cells (^*∗*^*P* < 0.05, ^*∗∗*^*P* < 0.01, ^*∗∗∗*^*P* < 0.001). (d) A549 cells showed a significant decrease in their proliferative and clonal ability after treating with YQYJ for 24 hours (^*∗∗∗*^*P* < 0.001).

We chose human lung cancer cells A549 to explore the pharmacodynamics mechanism of YQYJ. First, the inhibitory effect of YQYJ was evaluated by MTT on cell viability. Cells were treated with YQYJ at different concentrations (0.18, 0.25, 0.375, 0.5, 0.75, 1.0, 1.5, and 3.0 mg/mL). The results showed that YQYJ significantly inhibited A549 cell viability in a dose- and time-dependent manner (all *P* < 0.05) (Figures 7(a) and 7(b)). Kaempferol (10, 20, and 40 *μ*mol/mL) and quercetin (10, 20, and 40 *μ*mol/mL) were also effective in inhibiting A549 cell proliferation (all *P* < 0.05) (Figure 7(c)).

Next, we used the cell clone formation rate to evaluate the proliferative capacity of A549 cells. The low concentration of YQYJ (0.25 mg/mL) could effectively inhibit the proliferative capacity of A549 cells (*P* < 0.001) (Figure 7(d)).

### 3.4. YQYJ Regulates the Cell Cycle and Promotes Apoptosis

**Figure 8:**
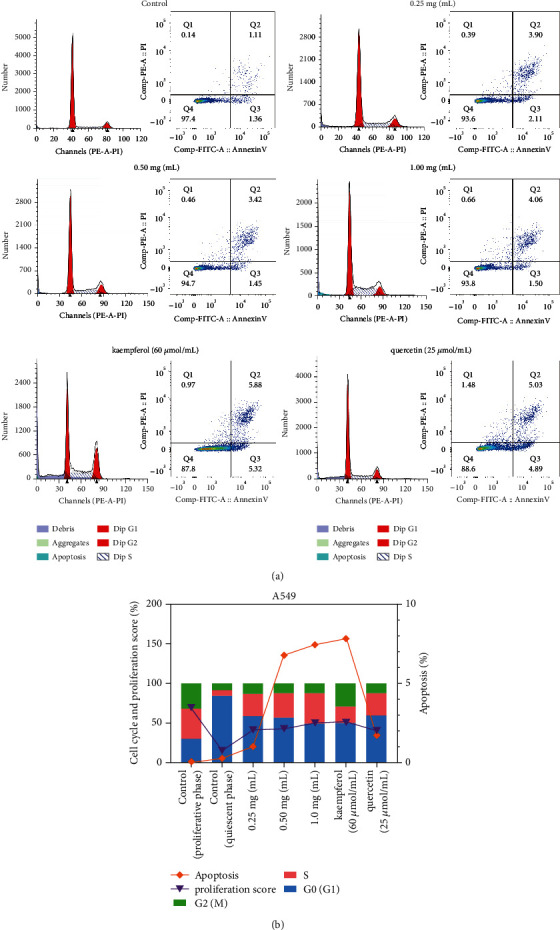
Flow cytometry results. (a) Cycle fitting map and apoptosis map of A549 cells; (b) the results of the cell cycle, apoptosis, and proliferation score of A549 cells.

Based on the previous network pharmacological analysis and the functional analysis of the core targets, we assumed that the regulatory effect of the YQYJ on the NSCLC cell cycle was important in the mechanism of action of YQYJ in the inhibition of NSCLC (Figures 3 and 4). In particular, it had an important role in the G1/S and G2/M transition of the mitotic cell cycle (Figure 5). We treated A549 cells with YQYJ, kaempferol, and quercetin for 24 hours and examined their cell cycle changes using PI or Annexin V/PI staining and flow cytometry. The quiescent phase A549 cells could be promoted by YQYJ from G0/G1 phase to the proliferation and division phase and blocked in the S and G2/M phases (*P* < 0.05) (Figure 8(b), Table 4). Compared to A549 cells in the proliferative phase, the G0/G1 phase of experimental groups was significantly upregulated (*P* < 0.05), and the proliferation score was significantly reduced (*P* < 0.05) (Figure 8(b), Table 4). Meanwhile, both kaempferol and YQYJ (0.5 mg and 1.0 mg/mL) were effective in promoting apoptosis in A549 cells (*P* < 0.05) (Table 4). The regulation of apoptosis by YQYJ was also confirmed by Zheng et al. [33], thus suggesting that YQYJ could effectively inhibit different proliferation periods of A549 cells.

### 3.5. Regulation of Potential Targets

**Figure 9:**
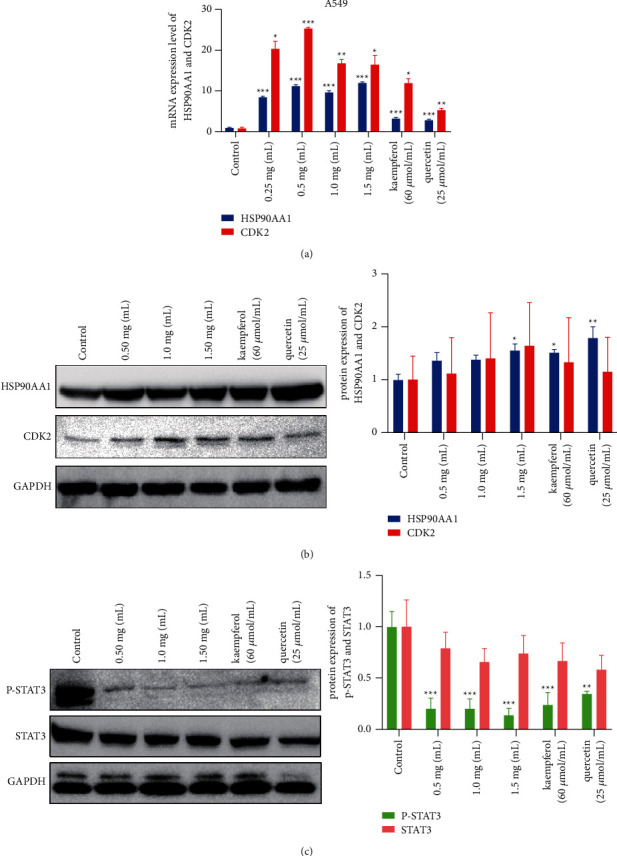
Regulation of YQYJ on A549 cells. (a) The mRNA expression levels of CDK2 and HSP90AA1. They were upregulated significantly by YQYJ, kaempferol, and quercetin (^*∗*^*P* < 0.05, ^*∗∗*^*P* < 0.01, ^*∗∗∗*^*P* < 0.001). (b) The protein expression level of HSP90AA1 and CDK2. It shows an upregulation trend in each group, especially in the YQYJ (1.5 mg/mL) group (^*∗*^*P* < 0.05), the kaempferol group (^*∗*^*P* < 0.05), and the quercetin group (^*∗∗*^*P* < 0.01). (c) The protein expression level of STAT3 and p-STAT3. YQYJ, kaempferol, and quercetin could downregulate protein phosphorylation significantly (^*∗∗*^*P* < 0.01, ^*∗∗∗*^*P* < 0.001).

In NSCLC, HSP90AA1 and CDK2 expression levels were generally lower than in normal tissues (Figures 6(a) and 6(b)). Also, the mRNA expression level of HSP90AA1 and CDK2 was upregulated observably by YQYJ, kaempferol, and quercetin (all *P* < 0.05) (Figure 9(a)). As shown in Figure 9(b), the protein expression levels of HSP90AA1 and CDK2 had an upregulation trend in experimental groups, especially in the YQYJ (1.5 mg/mL) group (*P* < 0.05), the kaempferol group (*P* < 0.05), and the quercetin group (*P* < 0.01). This confirmed the ability of active components to bind to the core targets, which is consistent with the molecular docking results (Table 2).

In addition, STAT3 and p-STAT3 have an important role in tumors [34]. STAT3 phosphorylation regulates cancer metastasis [35] and may be used as a biomarker of poor prognosis in lung cancer [36]. In this study, we explored the expression changes of STAT3 and p-STAT3 in A549 cells. The obtained results showed that YQYJ, kaempferol, and quercetin could all downregulate the STAT3 and p-STAT3 protein expression, especially in the YQYJ group, kaempferol group, and quercetin group of p-STAT3 (*P* < 0.05) (Figure 9(c)). These findings suggested that YQYJ may be important for regulating protein phosphorylation, inhibiting tumor progression, and improving prognosis.

## 4. Discussion

YQYJ is a TCM prescription used for the treatment of lung cancer. Clinical studies have confirmed that YQYJ can reduce chemotherapy-related toxic and side effects and improve the quality of life [2–4, 37]. Although some studies have explored the role of YQYJ on NSCLC, the exact mechanism of action remains unclear. To the best of our knowledge, the present study is the first that systematically illustrated the active components and targets of YQYJ and the potential core mechanism of YQYJ inhibiting NSCLC.

Network pharmacology results indicated that YQYJ had 89 active components and 544 potential targets. Its mechanism of action involves 21 signaling pathways (Table S1) and several biological processes, such as cellular processes, metabolism, and microenvironment information processing (Figure 2), including EGFR, JAK2, MET, CCND1, E2F1, STAT3, TP53, beta-sitosterol, tetrandrine, kaempferol, flavone, and quercetin. Functionally, YQYJ mainly acts on cellular components such as protein kinase complex, RNA polymerase II transcriptional regulatory complex, membrane microdomain, caveola, and so on. For example, CAV1, as a major protein component of caveola, has an important regulatory role in tumorigenesis, while highly expressed caveolin-1 was identified as an independent prognostic risk factor for NSCLC [38]. Knockdown of caveolin-1 inhibited the invasion and migration of lung cancer cells [38] and increased therapeutic sensitivity of lung cancer to cisplatin-induced apoptosis [39].

Next, we further analyzed 68 core targets of the YQYJ-NSCLC core network. The results showed that the regulatory effect of YQYJ on NSCLC was mainly reflected in regulating cell proliferation and death, promoting apoptosis, and regulating phosphorylation of target proteins (Figures 3(a) and 3(b)). In this network, many active components were found to bind one or more genes that contribute to proliferation and apoptosis. Molecular docking results demonstrated the good binding efficiency of this active component to core targets (HSP90AA1 and CDK2). Previous studies have clarified that the tumor weight and cancer cell proliferation index of C57 mice were significantly reduced by YQYJ decoction [33], and the VEGF expression of Lewis cells was significantly decreased [40]. Wang et al. [17] found that YQYJ could regulate the proliferation and apoptosis of A549 cells by reducing the amount of lactic acid produced by glycolysis products of lung cancer cells. Also, it was related to depressing the expression of HIF-1*α* and 6-phosphofructo-2-kinase/fructose-2,6-biphosphatase 3 (PFKFB3).

In the present study, YQYJ significantly inhibited the proliferation of A549 cells, promoted the apoptosis of A549 cells, and promoted A549 cells from the quiescent phase into the proliferative phase. The increase of cells in the proliferative phase helps to enhance the sensitivity of A549 cells to YQYJ. And then, the A549 cells were blocked in both S and G2/M phases which increased the apoptosis ratio (Figure 8, Table 4). The proliferation score of A549 cells treated with YQYJ was significantly reduced compared to A549 cells in the proliferative phase (Table 4). This suggested that YQYJ could promote death and inhibit the proliferation of NSCLC cells. Zheng et al. [33] found the regulation of proliferation score of C57 mice tumor by YQYJ, and Wang et al. [17] found that YQYJ could regulate the proliferation and apoptosis of A549 cells by increasing the dose of YQYJ. In addition, we found a downregulating trend in the transcriptomic data of NSCLC, although the overexpression of HSP90AA1, CDK2, and E2F1 was important for the proliferation of multiple tumors [29, 31, 41]. In the present study, the RT-qPCR and Western blot experiment confirmed that YQYJ upregulated the expression level of HSP90AA1 and CDK2. Enrichment results showed that the functions of CDK2 and HSP90AA1 were also related to the G2/M transition of the mitotic cell cycle, cell cycle regulation, DNA replication, and similar. To sum up, these results revealed that YQYJ has an inhibitory role in inhibiting NSCLC primarily by inhibiting cell proliferation and promoting apoptosis. This effect was also achieved through a variety of targets and signaling pathways.

The discovery of the relationship between the potential targets of YQYJ and the inhibition action of NSCLC, such as HSP90AA1 and CDK2, helps guide further research of the core targets and YQYJ. For instance, the inhibitors of CDK4/6 have been approved for the treatment of breast cancer [29], and inhibitors of HSP90 (geldanamycin (GA), radicicol (RD), and its semisynthetic derivatives) were in preclinical research of tumor [41]. Our results, the upregulation expression of HSP90AA1 and CDK2 was related to the inhibited action of NSCLC cells, confirmed the important role of the core targets in lung cancer and may provide new ideas for the related research on guiding the clinical treatment of lung cancer.

However, given the limitations of network pharmacological analysis and studies of Chinese natural medicine, core mechanistic studies of NSCLC inhibition by YQYJ have not yet included all pharmaceutical components and potential targets of action, which need to be further addressed by future pharmacological studies and clinical research. With the exploration of Chinese herbal medicine components and the study of NSCLC-related genes, it is expected to find more potential targets of YQYJ, which could explain the mechanism of action of YQYJ.

## 5. Conclusion

This is the first study that elaborated on the core network of YQYJ in the inhibition of NSCLC and its mechanism of action from the perspective of network pharmacology. We confirmed that YQYJ mainly inhibited the progression of NSCLC proliferation by regulating the process of genetic information replication and cell cycle progression during the development of lung cancer. In addition, we identified several miRNA and transcription factors associated with HSP90AA1 and CDK2 and explored the potential of HSP90AA1 and CDK2 as therapeutic targets for NSCLC, thus providing a direction and scientific basis for future studies of mechanisms of action.

## Figures and Tables

**Table 1:** The top 5 active components and targets in the core network of YQYJ inhibiting NSCLC.

Category	Gene name/Component name	Degree	Betweenness centrality	Closeness centrality
Component	Quercetin (MOL000098)	105	0.317593	0.489362
Component	Luteolin (MOL000006)	25	0.141721	0.440613
Component	Gamatin (479-85-6)	21	0.149231	0.415162
Component	Tetrandrine (518-34-3)	21	0.165844	0.403509
Component	Kaempferol (MOL000422)	20	0.034529	0.395189
Target	HSP90AA1	36	0.211704	0.469388
Target	CDK2	24	0.148645	0.473251
Target	PIK3CG	22	0.082792	0.444015
Target	TOP2A	19	0.164112	0.387205
Target	MAPK14	13	0.026462	0.353846

**Table 2:** Molecular docking of the core target proteins.

Target	CDK2 (kcal/mol)	HSP90AA1 (kcal/mol)
Gamatin	−7.94	−7.39
Kaempferol	−8.15	−7.77
Luteolin	−7.76	−7.36
Quercetin	−8.26	−8.24
Tetrandrine	−6.98	−7.01
Gemcitabine	−7.22	−7.25

**Table 3:** The miRNA and transcription factor-target networks of HSP90AA1 and CDK2 in NSCLC (LinkedOmics).

Enriched category	Targets	LUAD			LUSC		
		Geneset	Leading edge num	FDR	Geneset	Leading edge num	FDR
miRNA target	HSP90AA1	TAATGTG, MIR-323	59	0	CAGGGTC, MIR-504	37	0.072108
miRNA target	HSP90AA1	ATGTTAA, MIR-302C	73	0	GGGGCCC, MIR-296	31	0.072108
miRNA target	HSP90AA1	ATCATGA, MIR-433	42	0	GAGCCTG, MIR-484	44	0.10816
miRNA target	HSP90AA1	TTTGCAG, MIR-518A-2	79	0.001119	GACAATC, MIR-219	34	0.13298
miRNA target	HSP90AA1	TACTTGA, MIR-26A, MIR-26B	84	0.0012789	AGCGCTT, MIR-518F, MIR-518E, MIR-518A	5	0.14078
miRNA target	CDK2	ACGCACA, MIR-210	2	0.6621	AGGAAGC, MIR-516-3P	24	0.53934
miRNA target	CDK2	GACAATC, MIR-219	28	0.73015	GTGGTGA, MIR-197	17	0.54347
miRNA target	CDK2	TTCCGTT, MIR-191	6	0.764	TCCAGAG, MIR-518C	39	0.5539
miRNA target	CDK2	CTCAGGG, MIR-125B, MIR-125A	40	0.77051	ATATGCA, MIR-448	52	0.55946
miRNA target	CDK2	AGCGCAG, MIR-191	5	0.77057	GGTGTGT, MIR-329	38	0.57271
Transcription factor target	HSP90AA1	V$E2F1_Q6	93	0	V$E2F4DP1_01	82	0
Transcription factor target	HSP90AA1	V$E2F_02	91	0	V$E2F1_Q6	85	0
Transcription factor target	HSP90AA1	V$E2F1DP1_01	90	0	V$E2F_Q6	75	0
Transcription factor target	HSP90AA1	V$E2F1DP2_01	90	0	V$E2F_Q4	70	0
Transcription factor target	HSP90AA1	V$E2F4DP2_01	90	0	V$E2F_02	79	0
Transcription factor target	CDK2	V$E2F1_Q6	100	0	V$E2F_Q6	91	0
Transcription factor target	CDK2	V$E2F_Q6	86	0	V$E2F_Q4	90	0
Transcription factor target	CDK2	V$E2F_Q4	86	0	V$E2F1_Q6	93	0
Transcription factor target	CDK2	V$E2F4DP1_01	88	0	V$E2F_02	90	0
Transcription factor target	CDK2	V$E2F_02	87	0	V$E2F1DP1RB_01	83	0

**Table 4:** The result of the cell cycle, apoptosis, and proliferation score.

	Control (proliferative phase)	Control (quiescent phase)	0.25 mg	0.5 mg	1.0 mg	Kaempferol (60 *µ*M)	Quercetin (25 *µ*M)
G0/G1	30.56 ± 14.25	84.63 ± 3.011	58.75 ± 6.604^*∗∗*^^##^	57.38 ± 6.374^*∗∗*^^##^	49.82 ± 3.657^*∗∗*^^###^	49.29 ± 6.603^*∗*^^###^	59.41 ± 7.960^*∗∗∗*^^##^
S	37.60 ± 2.298	7.61 ± 1.953	28.51 ± 5.069^###^	30.68 ± 5.749^###^	38.24 ± 1.659^###^	21.71 ± 8.487^*∗∗*^^##^	28.07 ± 7.708^*∗*^^###^
G2/M	31.83 ± 15.975	7.76 ± 1.083	12.74 ± 1.544^*∗∗*^	11.95 ± 0.641^*∗∗*^	11.95 ± 1.996^*∗∗*^	29.00 ± 2.267^##^	12.52 ± 0.423^*∗∗*^
Apoptosis	0.05 ± 0.04	0.26 ± 0.148	1.03 ± 0.775	6.80 ± 5.218^*∗∗*^^##^	7.46 ± 2.262^*∗∗*^^##^	7.82 ± 3.385^*∗∗*^^##^	1.73 ± 0.444
Proliferation score	69.43 ± 14.252	15.36 ± 3.014	41.25 ± 6.610^*∗∗*^^##^	42.63 ± 6.372^*∗∗*^^##^	50.18 ± 3.652^*∗∗*^^###^	50.71 ± 6.504^*∗∗*^^###^	40.59 ± 7.960^*∗∗∗*^^###^

^*∗*^Experimental groups VS control (proliferative phase) group, ^*∗*^*P* < 0.05, ^*∗∗*^*P* < 0.01, ^*∗∗∗*^*P* < 0.001. # Experimental groups VS control (quiescent phase) group, ^#^*P* < 0.05, ^##^*P* < 0.01, ^###^*P* < 0.001.

## Data Availability

All relevant data are available and could be provided upon request to the corresponding author.

## Supplementary Materials

Supplementary Materials Supplementary Information S1: Preparation method of YQYJ lyophilized powder and experimental details of RT-qPCR and Western blot. Figure S1: The details of 89 active components and potential targets of NSCLC. Table S1: The details of the 21 signaling pathways of YQYJ.
