# Supplementary material for: The Core Mechanism of Yiqi Yangjing Decoction Inhibiting Nonsmall-Cell Lung Cancer
Source: Evid Based Complement Alternat Med. 2022 May 9;2022:2256671. doi: 10.1155/2022/2256671 (PMC9110163; doi:10.1155/2022/2256671)
Supplement: Supplementary Materials — Supplementary Information S1: Preparation method of YQYJ lyophilized powder and experimental details of RT-qPCR and Western blot. Figure S1: The details of 89 active components and potential targets of NSCLC. Table S1: The details of the 21 signaling pathways of YQYJ. [file 2256671.f1.zip › 2256671.f1/Figure S1.docx]

**
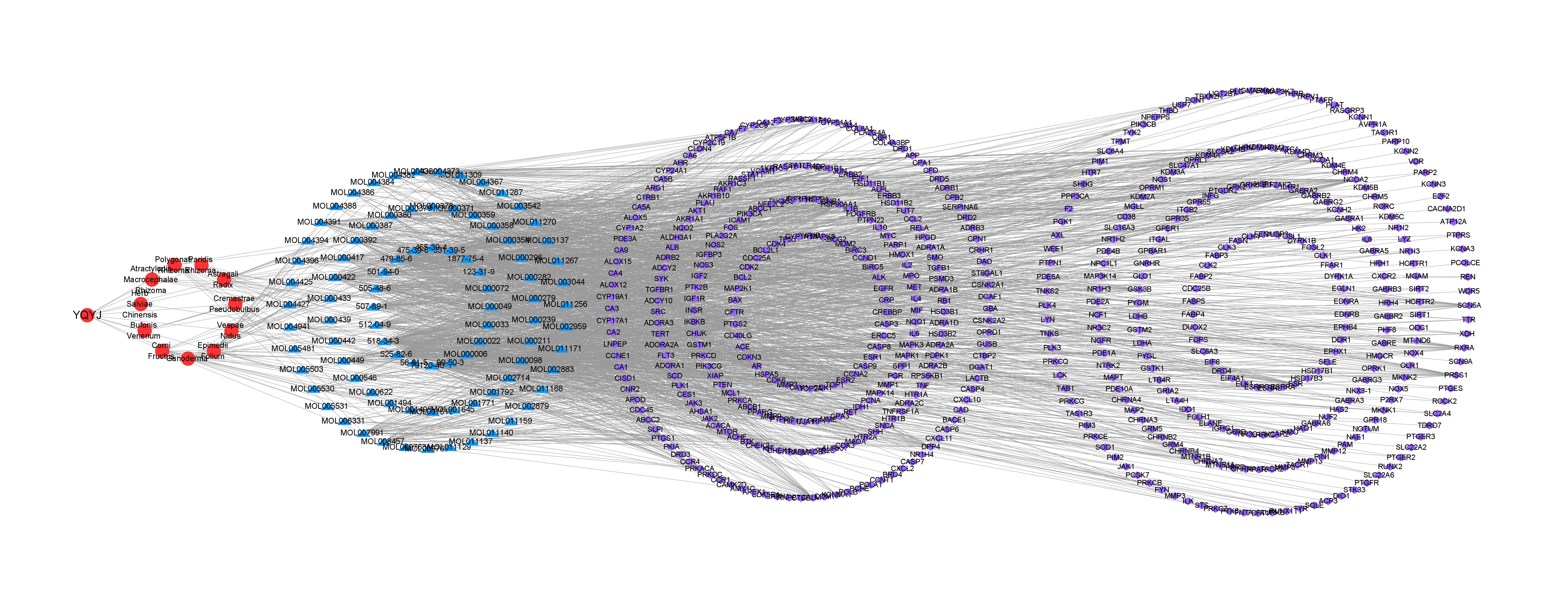
**

Figure S1：The details of 89 active components and potential targets of NSCLC. The red ellipse node represents traditional Chinese medicine YQYJ, the blue triangle node represents active compounds, and the purple circles delineate targets.
