# Supplementary figures and images for: The Core Mechanism of Yiqi Yangjing Decoction Inhibiting Nonsmall-Cell Lung Cancer
Source: Evid Based Complement Alternat Med. 2022 May 9;2022:2256671. doi: 10.1155/2022/2256671 (PMC9110163; doi:10.1155/2022/2256671)

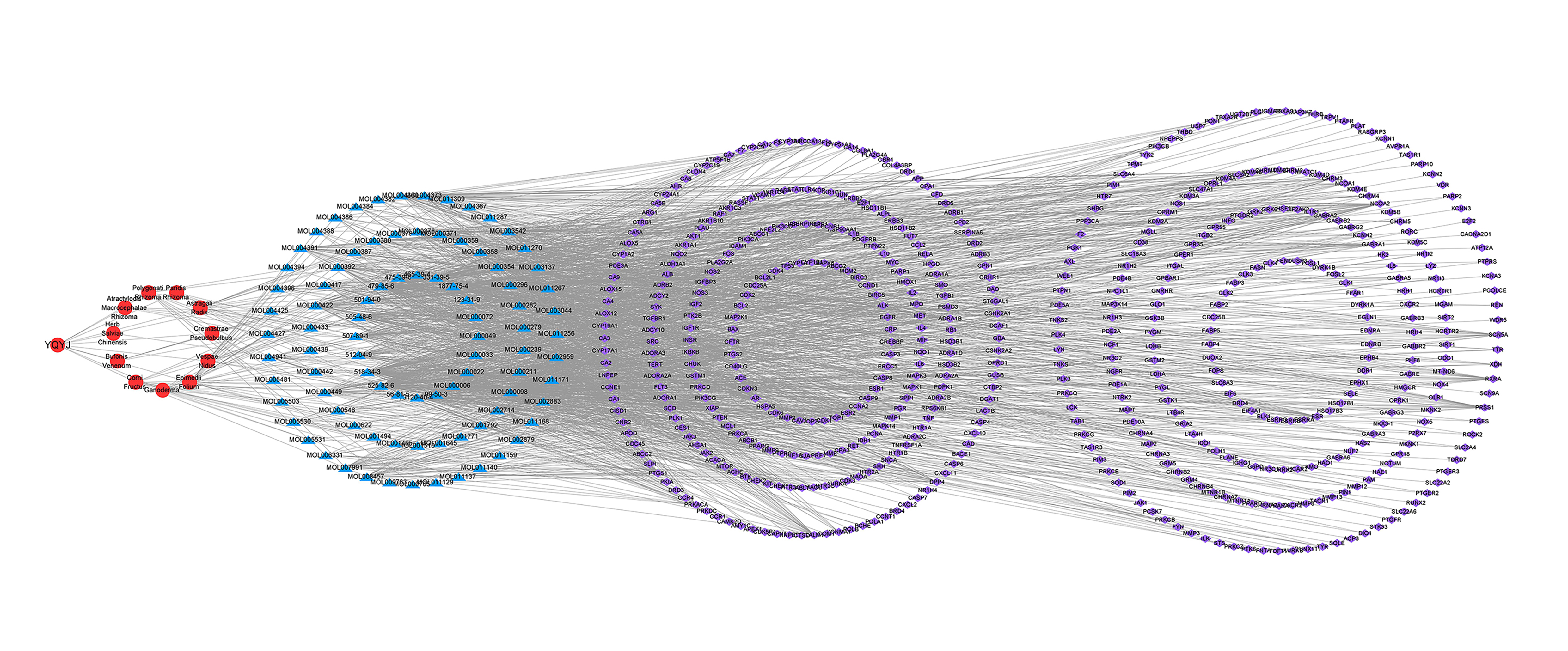

Supplement: Supplementary Materials — Supplementary Information S1: Preparation method of YQYJ lyophilized powder and experimental details of RT-qPCR and Western blot. Figure S1: The details of 89 active components and potential targets of NSCLC. Table S1: The details of the 21 signaling pathways of YQYJ. [file 2256671.f1.zip › 2256671.f1/Figure S1.jpg]
