# Supplementary material for: The Core Mechanism of Yiqi Yangjing Decoction Inhibiting Nonsmall-Cell Lung Cancer
Source: Evid Based Complement Alternat Med. 2022 May 9;2022:2256671. doi: 10.1155/2022/2256671 (PMC9110163; doi:10.1155/2022/2256671)
Supplement: Supplementary Materials — Supplementary Information S1: Preparation method of YQYJ lyophilized powder and experimental details of RT-qPCR and Western blot. Figure S1: The details of 89 active components and potential targets of NSCLC. Table S1: The details of the 21 signaling pathways of YQYJ. [file 2256671.f1.zip › 2256671.f1/SUPPLE~1.DOC]

**Supplementary Information S1**

1. **The preparation method of YQYJ lyophilized powder**

Chinese Herbal Medicine YQYJ contains Astragali Radix 30g, Ganoderma 15g, Atractylodis Macrocephalae Rhizoma 9g, Herb Salviae Chinensis 30g, Epimedii Folium 15g, Corni Fructus 15g, Cremastrae Pseudobulbus 15g, Polygonati Rhizoma 30g, Vespae Nidus 9g, Paridis Rhizoma 9g and Bufonis Venenum 6g. YQYJ (183g) was placed in 2000 milliliters of pure water, boiled for 60min, and then filtered with four layers of gauze. The filtrate was freeze-dried at -70℃, resulting in a powder of 36.5g. The YQYJ was dissolved in phosphate-buffered saline (PBS) (15 mg/ml), then stored at four °C as a stock solution. Most importantly, the drug solution was filtered by a filter membrane of 0.45μm and 0.22μm before each use.

2. **Experimental details of RT-qPCR**

Table 1: Primer Sequences of HSP90AA1, CDK2, and GAPDH

| **Primer Name** | Sequences (5' to 3') | **Purification method** |
| --- | --- | --- |
| homo CDK2 forward primer | CCTGGGCTGCAAATATTATTCC | PAGE |
| homo CDK2 reverse primer | TGGCTTGTAATCAGGCATAGAA | PAGE |
| homo HSP90AA1 forward primer | CCAGTTCGGTGTTGGTTTTTAT | PAGE |
| homo HSP90AA1 reverse primer | CAGTTTGGTCTTCTTTCAGGTG | PAGE |
| homo GAPDH forward primer | ACAACTTTGGTATCGTGGAAGG | PAGE |
| homo GAPDH reverse primer | GCCATCACGCCACAGTTTC | PAGE |

**Approach：**

(1) Total RNA extraction (E.Z.N.A.® Total RNA kit, Omega Bio-Tek (Norcross, Georgia, USA)

ⅰ. Disrupt cells (5 x 10^6^ cells) with 350*μ*L GTC Lysis Buffer (containing 20 *μ*L 2-mercaptoethanol/mL). Vortex or pipet up and down to mix thoroughly.

ⅱ. Homogenize the cells accordingly to one of the following methods:

Homogenizer Mini Column (HCR003): Load the lysate into a Homogenizer Mini Column pre-inserted into a 2 ml Collection Tube. Centrifuge at maximum speed for two minutes to collect the homogenized lysate.

ⅲ. Insert a HiBind DNA Mini Column into a 2 ml Collection Tube. Transfer the homogenized lysate to the HiBind DNA Mini Column, and centrifuge at 13 000 XG for 1 minute.

ⅳ. The buffer in the 2ml collection tube was added with 500 μl of anhydrous ethanol for 15s. And then, the mixture was added to a HiBind@RNA centrifugation column and centrifuged at 10,000 XG for 1 minute.

ⅳ. The 500ul RWC wash solution was added to the RNA centrifugation column and centrifuged at 10,000 XG for 1 minute.

ⅴ. Two washes at 500ul RNA wash buffer Ⅱ (10,000 XG 1 minute centrifugal cells) can be centrifuged again for 2 minutes to dry the RNA tubes.

ⅵ. The RNA column was transferred to a 1.5mL EP tube and the RNA eluted with 40 to 70*μL* DEPC of water was made sure to add to the central position and was centrifuged at 10,000 XG for 1 minute.

(2) Reverse transcription (HiScript Ⅱ Reverse Transcriptase, Vazyme (Nanjing, China))

Added 5 HiScript Ⅱ select mix 4*μL*, Random hexamers (50 ng/ml) 1*μL*, total RNA *<*1*μ*g, and add ddH2O to 20ul. Reversed it on the PCR amplicon at 50℃ for 15 minutes and 85℃ for 5 seconds.

(3) PCR amplified reaction (ChamQ® SYBR qPCR Master Mix, Vazyme (Nanjing, China))

Table 2: The Reaction system

| Reagents | Volume (*μL*) |
| --- | --- |
| 2×chamQ SYBR Color qPCR Master mix | 5 |
| Upstream primers (10 *μ*mol) | 0.2 |
| Downstream primers (10 *μ*mol) | 0.2 |
| 50× ROX (ⅰ) Predenaturation: | 0.2 |
| Template cDNA | 1 |
| ddH2O | To 10 |

(ⅰ) Predenaturation: reps 1, 95℃ 30 sec.

(ⅱ) Circular reaction: reps 40, 95℃ for 10 sec, and 60℃ for 30 sec.

(ⅲ) Melting curve: reps 1, 95℃ for 15 sec, 60℃ for 60 sec, and 95℃ for 15 sec.

3. **Experimental details of Western blot.**
(1) Protein sample preparation:

Total protein from each experimental group was removed using RIPA buffer (P0013B, Beyotime, Shanghai, China). The protein concentration was then detected using BCA Protein Concentration Assay Kit (P0010, Beyotime, Shanghai, China).

(2) SDS-PAGE electrophoresis

According to the reagent instructions, prepared 10% separation glue and 5% Concentrated glue. The solution volume of 20 *μ*g protein was the upper sample volume and denatured by cooking at 100℃ for 10 minutes before the loading. And then, the protein was isolated at 80V for 30 minutes and 120V for 60 to 90 minutes until bromoophlan just ran out.

(3) Transfer

The transfer standard was 200 mA for 120 min using the 0.22*μ*m [pall](https://cn.bing.com/dict/search?q=pall&FORM=BDVSP6&cc=cn) [membrane](https://cn.bing.com/dict/search?q=membrane&FORM=BDVSP6&cc=cn).

(4) Immunoreaction

After blocking with a blocking solution containing 5% skim milk for one hour at room temperature, a primary antibody (volume dilution ratio of 1:1000) was added, reacted at 4℃ overnight, and rinsed three times with TBST. Then the horseradish peroxidase-labeled IgG secondary antibody (volume dilution ratio of 1:1000)was added and reacted for two hours at room temperature.

(5) Chemiluminescence

The chemiluminescence was performed according to the instructions of the ECL kit and photographed by the gel imaging system.

(6) Image analysis

Experimental images were analyzed using Image J, and statistical analysis of experimental data was performed using GraphPad Prism 8.0.2. Geometric data are described in x ± s. The data of each group were tested for normality, and the group was compared before and after itself. Those who meet the normal distribution used paired t-test, and the rank-sum test for paired measurement data comparison was used. The one-way ANOVA and a rank-sum test were used for multiple independent sample comparisons. *P* *<* 0.05 indicates a statistically significant difference.

Table 3 The details of antibodies.

| Name | Compony | number | lot number |
| --- | --- | --- | --- |
| STAT3[EPR787Y] | ABCAM | ab68153 | GR3220487-9 |
| phospho STAT3(Y705) | ABCAM | ab76315 | GR287047-50 |
| Anti-rabbit IgG HRP-linked antibody | CST | 7074S | 30 |
| HSP90α(D1A7) Rabbit mAb | CST | 8165 | 1 |
| CDK2(78B2) Rabbit mAb | CST | 2546 | 8 |
