# Supplementary material for: The Core Mechanism of Yiqi Yangjing Decoction Inhibiting Nonsmall-Cell Lung Cancer
Source: Evid Based Complement Alternat Med. 2022 May 9;2022:2256671. doi: 10.1155/2022/2256671 (PMC9110163; doi:10.1155/2022/2256671)
Supplement: Supplementary Materials — Supplementary Information S1: Preparation method of YQYJ lyophilized powder and experimental details of RT-qPCR and Western blot. Figure S1: The details of 89 active components and potential targets of NSCLC. Table S1: The details of the 21 signaling pathways of YQYJ. [file 2256671.f1.zip › 2256671.f1/Table S1.doc]

Table S1：The details of the 21 signaling pathways of YQYJ.

| ID | Description | pvalue | p.adjust | qvalue | Count |
| --- | --- | --- | --- | --- | --- |
| hsa04020 | Calcium signaling pathway | 2.74E-17 | 6.17E-16 | 2.37E-16 | 47 |
| hsa04066 | HIF-1 signaling pathway | 3.26E-17 | 6.82E-16 | 2.62E-16 | 35 |
| hsa04668 | TNF signaling pathway | 8.58E-17 | 1.68E-15 | 6.44E-16 | 35 |
| hsa04064 | NF-kappa B signaling pathway | 9.01E-14 | 8.51E-13 | 3.27E-13 | 30 |
| hsa04010 | MAPK signaling pathway | 2.12E-13 | 1.83E-12 | 7.02E-13 | 53 |
| hsa04068 | FoxO signaling pathway | 6.57E-13 | 4.94E-12 | 1.90E-12 | 33 |
| hsa04071 | Sphingolipid signaling pathway | 1.28E-12 | 9.36E-12 | 3.60E-12 | 31 |
| hsa04151 | PI3K-Akt signaling pathway | 2.93E-12 | 2.00E-11 | 7.68E-12 | 57 |
| hsa04024 | cAMP signaling pathway | 5.42E-12 | 3.61E-11 | 1.39E-11 | 42 |
| hsa04012 | ErbB signaling pathway | 7.48E-11 | 3.92E-10 | 1.51E-10 | 24 |
| hsa04370 | VEGF signaling pathway | 7.48E-11 | 3.92E-10 | 1.51E-10 | 20 |
| hsa04022 | cGMP-PKG signaling pathway | 2.98E-09 | 1.25E-08 | 4.79E-09 | 32 |
| hsa04014 | Ras signaling pathway | 3.18E-08 | 1.16E-07 | 4.47E-08 | 37 |
| hsa04072 | Phospholipase D signaling pathway | 5.61E-07 | 1.75E-06 | 6.72E-07 | 26 |
| hsa04630 | Jak-STAT signaling pathway | 1.04E-05 | 2.68E-05 | 1.03E-05 | 25 |
| hsa04015 | Rap1 signaling pathway | 1.90E-05 | 4.75E-05 | 1.83E-05 | 29 |
| hsa04152 | AMPK signaling pathway | 2.57E-05 | 6.21E-05 | 2.39E-05 | 20 |
| hsa04371 | Apelin signaling pathway | 5.87E-05 | 1.30E-04 | 5.01E-05 | 21 |
| hsa04150 | mTOR signaling pathway | 1.05E-04 | 2.27E-04 | 8.72E-05 | 22 |
| hsa04310 | Wnt signaling pathway | 5.38E-04 | 1.05E-03 | 0.000403962 | 21 |
| hsa04340 | Hedgehog signaling pathway | 0.028243463 | 0.045974081 | 0.01767281 | 7 |
